# Prognostic significance of a complete pathological response after induction chemotherapy in operable breast cancer

**DOI:** 10.1038/sj.bjc.6600210

**Published:** 2002-04-08

**Authors:** P Chollet, S Amat, H Cure, M de Latour, G Le Bouedec, M-A Mouret-Reynier, J-P Ferriere, J-L Achard, J Dauplat, F Penault-Llorca

**Affiliations:** Centre Jean Perrin, Bureau de Recherche Clinique, 58 Rue Montalembert, B.P.392, 63011 Clermont-Ferrand Cedex 1, France; INSERM U484,Rue Montalembert, 63005 Clermont-Ferrand Cedex, France

**Keywords:** pathological response, prognostic factor, breast cancer, neoadjuvant chemotherapy

## Abstract

Only a few papers have been published concerning the incidence and outcome of patients with a pathological complete response after cytotoxic treatment in breast cancer. The purpose of this retrospective study was to assess the outcome of patients found to have a pathological complete response in both the breast and axillary lymph nodes after neoadjuvant chemotherapy for operable breast cancer. Our goal was also to determine whether the residual pathological size of the tumour in breast could be correlated with pathological node status. Between 1982 and 2000, 451 consecutive patients were registered into five prospective phase II trials. After six cycles, 396 patients underwent surgery with axillary dissection for 277 patients (69.9%). Pathological response was evaluated according to the Chevallier's classification. At a median follow-up of 8 years, survival was analysed as a function of pathological response. A pathological complete response rate was obtained in 60 patients (15.2%) after induction chemotherapy. Breast tumour persistence was significantly related to positive axillary nodes (*P*=5.10^−6^). At 15 years, overall survival and disease-free survival rates were significantly higher in the group who had a pathological complete response than in the group who had less than a pathological complete response (*P*=0.047 and *P*=0.024, respectively). In the absence of pathological complete response and furthermore when there is a notable remaining pathological disease, axillary dissection is still important to determine a major prognostic factor and subsequently, a second non cross resistant adjuvant regimen or high dose chemotherapy could lead to a survival benefit.

*British Journal of Cancer* (2002) **86**, 1041–1046. DOI: 10.1038/sj/bjc/6600210 www.bjcancer.com

© 2002 Cancer Research UK

## 

Primary chemotherapy has become the standard treatment of inflammatory and locally advanced breast cancer (LABC) and has more recently been extended to the management of patients with operable disease, eligible for mastectomy, mainly in order to increase the rate of breast conservation. There are multiple aspects regarding this treatment modality that have not been sufficiently elucidated (De Lena *et al*, 1978; Hortobagyi *et al*, 1983; Lippman *et al*, 1986; Scholl *et al*, 1994; Fisher *et al*, 1997, 1998). The clinical response to neoadjuvant chemotherapy, which is commonly reported, does not always accurately reflect the pathological response: residual tumour is frequent in clinically complete responses, and conversely some complete pathological responses are found in good partial clinical responses. The residual *in situ* carcinoma has unprecised prognostic significance, and is taken into account only in some pathological classifications, as that of Chevallier *et al* (1993). It appears then of interest to study the clinical prognostic value of the pathological response on patient outcome.

Chemotherapy has been given in the adjuvant or neoadjuvant setting to destroy occult distant metastases and to improve the disease-free survival. The induction chemotherapy has over adjuvant treatment a triple advantage: (1) to allow an earlier systemic treatment, even before local care; (2) to increase breast conservation rate; (3) to give an individual evaluation of its efficacy. However, the true consequences of these advantages are still uncertain.

As for the third point, one can suppose that a complete response should reflect the chemosensitivity of occult distant metastatic sites; then patients who have a pathological complete response (pCR) in both the primary breast tumour and axillary lymph nodes after induction chemotherapy should have better overall and disease-free survival rates, compared with patients with poor responses. Currently, only a small number of studies have been published concerning the outcome of patients with a pathological complete response (pCR) of both the primary tumour and axillary lymph nodes after neoadjuvant chemotherapy (Feldman *et al*, 1986; Chevallier *et al*, 1993; Fisher *et al*, 1997, 1998; Pierga *et al*, 2000). Some clinical and biologic factors (nodal, status, age, stage, estrogens receptor, tumour size) have been analysed for the potential predictive significance of complete histologic clearance from both the breast and axillary lymph nodes after primary chemotherapy (Kuerer *et al*, 1999); patients with a pCR had initial tumours that were more likely to be oestrogens receptor-negative and anaplastic but of smaller size than those of patients with less than a pCR.

In the current study, we propose an analysis of 451 operable breast cancer patients who received neoadjuvant chemotherapy into five successive phase II trials from the same institution, designed to improve breast conservation and pCR rates with the available drugs in association. The construction of these trials was based on the association of ‘major’ drugs for breast cancer, i.e. giving a response rate of at least 40% at conventional dose in first metastatic line: one to three major drugs have been associated, also with less effective but highly employed drugs as fluorouracil, cyclophosphamide and methotrexate. However, Taxotere® alone has been tested, as it had a better clinical activity than doxorubicin at the optimal dose in first line for metastatic patients (Khayat *et al*, 2001). The pCR rates obtained have raised from 5.6 to 33.3%; all these individual studies have been published (Belembaogo *et al*, 1992; Van Praagh *et al*, 1995, 2001; Chollet *et al*, 1997, 2000). The database of individual survival has been actualised and pooled to explore the prognosis value of pCR on a large number of patients. Our goal was also to determine whether the pathological response in breast could be significantly related to axillary disease.

## PATIENTS AND METHODS

### Patient selection

Primary chemotherapy was indicated for operable breast cancer histologically or cytologically documented, of 30 mm in diameter or more, or situated in the central area of the nipple, and divided into stage IIA, IIB, IIIA, and some IIIB, using the TNM UICC (International Union against Cancer) (Sobin and Wittekind, 1997). The diagnosis was usually established by fine-needle aspiration or per cutaneous microbiopsy of the primary tumour and clinically involved axillary lymph nodes. Patients with primary inflammatory carcinoma or with a long clinical history of ‘neglected’ tumour in breast were excluded and offered enrolment onto other treatment protocols. Before treatment, a core biopsy with a ‘surecut’ needle confirmed the pathological variety and has been used to determine prognosis factors as hormonal receptors, pathological SBR grade and cell cycle parameters (cells in S phase, aneuploidy).

The staging work-up included a complete history and physical examination, complete blood cell and platelet counts, blood chemistry analysis, CEA and CA 15.3, electrocardiography, chest radiograph, abdominal ultrasonography, bone scan at presentation. The local evaluation comprised clinical and echographic measurement of tumour and nodes, a bilateral mammography, in some cases a breast IRM, and was repeated every two or three cycles of chemotherapy.

### Treatment modalities

Between 1982 and 2000, the 451 consecutive patients treated by primary chemotherapy for an operable breast cancer (median diameter: 40 mm) were registered into five prospective phase II trials of regimens containing:

anthracyclin-based regimens: AVCF/M, doxorubicin/Adriamycin®, vincristine, fluorouracil and cyclophosphamide plus or minus methotrexate (164 patients); NEM, vinorelbine/Navelbine®, epirubicin, methotrexate (112 patients); TNCF, theprubicin/THPadriamycin®, vinorelbine/Navelbine®, fluorouracil and cyclophosphamide (69 patients);
or a taxane alone: docetaxel/Taxotere® (86 patients);
or anthracyclin plus navelbine and taxane (paclitaxel/Taxol®): NET (20 patients).

Table 1

**Table 1:**
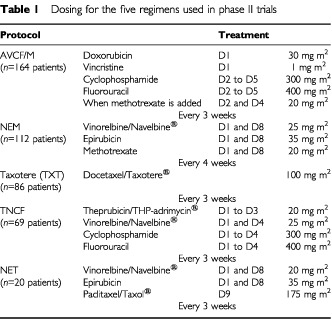
Dosing for the five regimens used in phase II trials

gives the precise protocols for the five regimens, which were administered for six cycles. The primary end points for the induction chemotherapy regimens consisted of determination of clinical and particularly pathological response rates, as reported previously (Belembaogo *et al*, 1992; Van Praagh *et al*, 1995, 2001; Chollet *et al*, 1997, 2000).

The patient was informed of the therapeutic choice, and from the TNCF protocol (i.e. for TNCF, Taxotere® and NET regimens) gave informed written consent, as being included in a prospective phase II trial according to the available new drugs at this date and to the results of previous studies. Each patient was then entered prospectively into the database and observed longitudinally. The complete medical records of all of the patients were available for review at the time of this analysis. Chemotherapy was administered at 21- to 28-day intervals. Patients were operated after six cycles by conservative surgery for good responders and modified radical mastectomy (MRM) for nonresponders.

Locoregional radiotherapy was instituted within 6 weeks after the completion of surgery or of chemotherapy, if adjuvant chemotherapy was given. Postoperative irradiation treatment was delivered to the chest wall, internal mammary lymph nodes, and supraclavicular/ axillary lymph nodes. In case of important residual disease, patients could receive additional courses of chemotherapy. Finally, menopausal patients with hormonal receptor-positive tumours received tamoxifen for 5 years. Five-year compliance with tamoxifen therapy was greater than 90%.

### Assessment of response

Clinical responses to neoadjuvant chemotherapy were classified by the following criteria: complete response (CR), a total resolution of the breast tumour and axillary adenopathy based on clinical, echographic and radiographic examinations; partial response (PR), a 50% or greater reduction of the product of the two largest perpendicular dimensions of the breast mass and axillary adenopathy; minor response (MR), a less than 50% reduction of the product of the two largest perpendicular dimensions of the breast mass and axillary adenopathy; no change in clinical status (NC); and progressive disease (PD). Patients had initially a tattooing point at the centre of tumour area to help the knowledge of the precise tumoural site in case of complete response.

We used the pathological classification of Chevallier *et al* (1993). The pathological response to neoadjuvant chemotherapy was classified as follows:

class I: no evidence of residual tumour in the breast or axillary lymph nodes,
class II: only residual *in situ* carcinoma,
class III: residual tumour evidently modified by treatment,
class IV: histologically unmodified tumour.

A minimum of 10 sections from the region of the initial primary tumour site was examined; pCR is usually considered as the sum of classes I and II.

### Follow-up and survival

During the first 5 years of follow-up, patients had a history and physical examination, complete blood count, liver function tests, serum CEA and CA 15–3 every 6 months. During the next 10 years, patients had only these clinical examination and biology every 6 months, and mammography performed at yearly intervals. At any time, if the patient exhibited elevated liver function tests, an abdominal computed tomographic scan or ultrasound of the liver was obtained. Overall survival (OS) and disease-free survival (DFS) were calculated from the date of diagnosis with Kaplan–Meier method (Kaplan and Meier, 1958); the cut-off date was May 22nd, 2001. The log-rank statistic was used for univariate comparisons of survival end points (Mantel, 1966); two-tailed results are reported. A stepwise Cox regression procedure was used to classify the pathological response among main prognostic factor (Cox, 1972). *P* of 0.05 or lower was considered statistically significant.

## RESULTS

Table 2

**Table 2:**
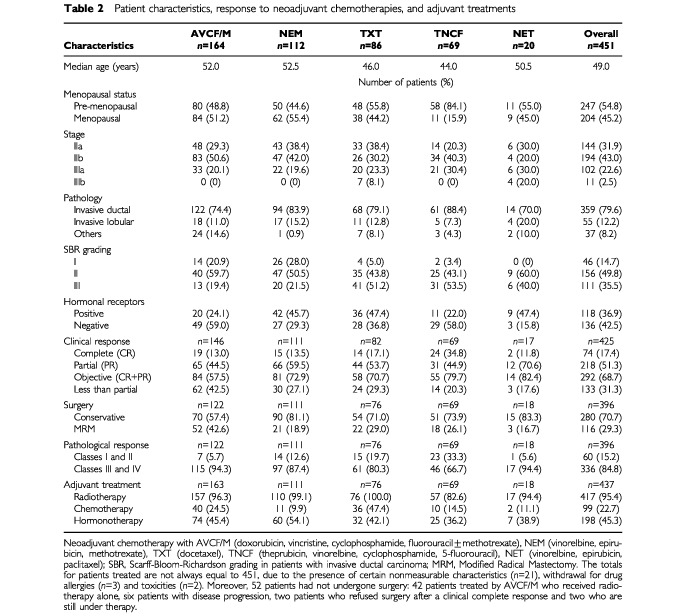
Patient characteristics, response to neoadjuvant chemotherapies, and adjuvant treatments

lists the pretreatment patient and tumour characteristics for the 451 patients. The median age was 49 years (range 25 to 80 years) and 247 out of the 451 patients (54.8%) were premenopausal. The median largest diameter of the primary tumour was 40 mm for the entire group of patients. There were six (1.3%) tumours of 20 mm or less [T1], 309 (68.5%) tumours more than 20 mm but no more than 50 mm [T2], 125 (27.7%) tumours more than 50 mm [T3], 11 (2.5%) tumours of any size with direct extension to chest wall or skin [T4]. Only 171 (37.9%) of the patients did not have clinically involved adenopathy [N0] at diagnosis. Three hundred and thirty-eight patients (74.9%) had a stage II disease [T1-2-3, N0-1, M0: no distant metastasis], 102 (22.6%) a stage IIIa [T2-3, N1-2, M0] and only 11 (2.5%) a stage IIIb [T4, N0-1, M0]. Pathological proof of microbiopsy gave 358 (79.6%) invasive ductal and 55 invasive lobular carcinomas, with 35.5% of SBR grade III tumours (*n*=111). Tumour oestrogens receptor status at diagnosis was determined before treatment in 325 patients (72.1%), and the progesterone receptor status was determined in 321 patients (71.2%).

### Tumour response to neoadjuvant chemotherapy

Because of technical reasons or incomplete treatments, 36 patients were inevaluable for response (18 AVCF/M, 1 NEM, 3 NET and 4 Taxotere®). Clinical response was consequently determined in 425 patients (Table 2). Two hundred and eighteen patients (51.3%) had at least a partial response and 74 (17.4%) a complete response, allowing an overall clinical response rate of 68.7% but the percentage was variable according to the relative potency of the treatment used.

Fifty-five patients could not be included for the pathological evaluation after six courses: 42 AVCF/M treated by radiotherapy alone (initially good responders were not immediately operated, they have been considered as invaluable for pathological response), three acute allergies to Taxotere®, six progressions, two surgery refusals after clinical complete response, two too early. Then, 396 patients underwent either a segmental mastectomy (*n*=280, 70.7%) or a modified radical mastectomy (*n*=116, 29.3%) after six cycles of chemotherapy; 277 (70.0%) patients had an axillary dissection after chemotherapy (Table 2).

According to the Chevallier classification after cases review, 40 patients had a class I, 20 a class II, 107 a class III and 229 a class IV, allowing a pCR rate of 15.2% (60 of the 396 operated patients); the percentage of pCR was also variable according to the effectiveness of the regimen used: from 5.6% with NET to 33.3% with the semi-intensive TNCF regimen. As adjuvant treatment, 417 patients received radiotherapy, 99 a second chemotherapy, and 198 received tamoxifen (Table 2).

### Pathological response and survival

**Figure 1:**
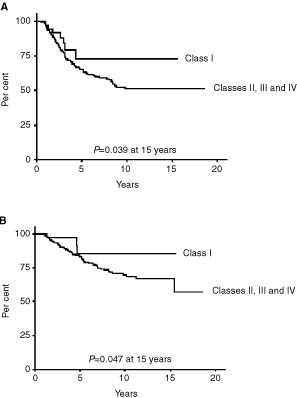
Analyse of the disease-free survival **(A)** and overall survival **(B)** as a function of pathological response after neoadjuvant chemotherapy. Pathological response was evaluated according to Chevallier's classification (Chevallier *et al*, 1993). Survival was analysed in patients with Class I response *vs* in patients with Classes II, III, IV responses.

The patients have been included from January 1982 to May 2000 and median follow-up reached 8 years as of May 22nd, 2001. The OS and DFS were analysed as a function of pathological response after neoadjuvant chemotherapy (Figure 1

). Survival was evaluated in patients with pCR (classes I and II) and was compared to nonresponders survival (classes III and IV). Similarly, survival of patients with class I response was compared with that of patients with classes II, III and IV responses.

Patients with pCR had an improved 15-year DFS compared with that of patients with incomplete tumour responses (classes III and IV responses) with a *P* value of 0.0053. When class I was considered alone against other classes, the difference was still significant (Figure 1A: log-rank; χ^2^=5.076, *P*=0.024). The difference observed between the responders and nonresponders was due to 35.4% relapses in the class III and IV group *vs* only 18.3% in patients with pCR.

Conversely, the 15-year OS was not significantly different in patients with pCR when it was compared with the group of patients with residual invasive tumours (classes III and IV), with a *P* value of 0.37. However, when class I was considered alone against other classes, the difference became significant (Figure 1B: log-rank; χ^2^=3.950, *P*=0.047). This appeared to be due to lesser results in the class II group. Conversely, OS in class I was about 85.1% at 15 years *vs* around 66.7% in the other classes.

The Cox regression analysis showed that prognostic factors of survival were node involvement (*P*<4.10^−3^) followed by SBR grade (*P*<0.04), and pathological type for DFS only (*P*=0.012). Pathological response did not seem to influence survival in multivariate analysis (*P*=0.11 for DFS and *P*=0.96 for OS).

### Pathological response and nodes involvement

Positive axillary nodes are the main prognostic factor in breast cancer; our goal was to determine whether the pathological response in breast was significantly related to axillary disease. The residual pathological size of the tumour at surgery was evaluated when possible, i.e. in 255 out of the 277 patients who underwent axillary dissection. On univariate analysis, the residual tumour size was found to be correlated with node involvement. The pathological response in breast was strongly correlated with pathological node status with a 3.1-fold increased relative risk of involvement for patients with remaining tumour in breast (*P*=5.10^−6^). Twenty-eight patients (80.0%), who had no residual tumour in breast at surgery, were negative for axillary disease compared with 85 patients (38.8%) in the nonresponders group. No significant correlation between the residual tumour size and prognosis was found (*P*=0.31).

## DISCUSSION

A pCR in the breast and axilla can be obtained in up to 33% of the cases, as a function of treatment used, in operable breast cancer (Hortobagyi *et al*, 1983; Lippman *et al*, 1986; Scholl *et al*, 1994; Schwartz *et al*, 1994; Powles *et al*, 1995; Brain *et al*, 1997; Bonadonna *et al*, 1998; Fisher *et al*, 1998; Morrell *et al*, 1998; Kuerer *et al*, 1999). It is known that neoadjuvant chemotherapy is able to convert clinically involved lymph nodes to a pathologically negative status in 25–38% of breast tumours. However currently, only a small number of studies have been published concerning the outcome of patients with a pCR of both the primary tumour and axillary lymph nodes after neoadjuvant chemotherapy (Feldman *et al*, 1986; Fisher *et al*, 1997, 1998; Kuerer *et al*, 1999; Pierga *et al*, 2000).

The results of our study indicate that in treating an operable breast cancer greater than 30 mm in diameter, neoadjuvant chemotherapy can completely clear the breast and axillary lymph nodes of any microscopic evidence of invasive tumour, as assessed by standard histologic examination. This was observed in 60 out of 396 patients operated after chemotherapy (15.2%). The complete tumour clearance (pCR) must be considered in breast and axilla as a new prognosis factor with putative individual value, early postulated in the study of Feldman *et al* (1986). Only a few papers have been published concerning the incidence and outcome of patients with a pCR in the primary tumour and axillary lymph nodes after neoadjuvant chemotherapy (Machiavelli *et al*, 1998; Kuerer *et al*, 1999). Most of the literature concerning pCR rates after induction treatment refers to and reports on pCRs in the primary tumour alone (Schwartz *et al*, 1994; Morrell *et al*, 1998; Bonadonna *et al*, 1998). In the National Surgical Adjuvant Breast and Bowel Project B-18 trial (Fisher *et al*, 1997, 1998), 13 (7%) of the 185 operable patients with clinically positive axillary lymph nodes treated with neoadjuvant chemotherapy were found to have no residual invasive tumour in the breast and axillary tissue upon pathological examination. However, pCR was documented only in clinically complete responses and, it is known that it can be found in a proportion of good partial clinical responses.

Does a pCR after chemotherapy simply identify patients who have a biologically predetermined excellent prognosis, or can the early initiation of systemic therapy alter the course of the disease, as compared with conventional primary surgical intervention followed by chemotherapy? Most trials of neoadjuvant chemotherapy followed by local therapy *vs* local therapy followed by postoperative chemotherapy did not show any survival advantage for patients who initially received chemotherapy (Schaake-Koning *et al*, 1985; Mauriac *et al*, 1991; Scholl *et al*, 1994; Powles *et al*, 1995; Fisher *et al*, 1997, 1998; Cunningham *et al*, 1998). Conversely, from the currently available data, it can be stated with some confidence that neoadjuvant chemotherapy does not bestow a survival disadvantage.

The principal goal of our study was to determine if the patients without any histologic evidence of residual invasive tumour, i.e. patients with pCR after neoadjuvant chemotherapy, had a survival better than patients with residual invasive tumour. Although a pCR was not synonymous with a definitive cure, it can predict at the individual level a more favourable outcome with a reduced relapse rate. These potential patients should be in partial response after four courses in order to spare them from additional ineffective and toxic treatment. Conversely, one of the most striking benefits of neoadjuvant chemotherapy could rather be the identification of patients with a minimal response than the early identification of patients with an excellent response to chemotherapy. This new prognostic factor offers the advantage of possible therapeutic change compared with the adjuvant treatment, which is ‘blind’ and only based on statistical parameters. Actually, the residual disease accounts for a worse prognosis and invites to perform clinical studies evaluating prospectively the eventual role of a non cross-resistant chemotherapy in those patients with unmodified or important residual tumour in breast and nodes.

Because neoadjuvant chemotherapy is often effective in reducing the size of the primary tumour and in downstaging the axilla from positive to negative status, we wanted to determine whether pathological response of the primary tumour to neoadjuvant chemotherapy could be significantly related to axillary disease. A residual tumour disease in breast is more frequently associated with positive nodes at axillary surgery (*P*=5.10^−6^). Thus, for responders (no residual tumour), the chance of having histologically negative nodes after neoadjuvant chemotherapy is very high (80.0%), regardless of the findings by clinical examination at diagnosis. In patients with residual tumour at surgery, the response lack of the primary tumour coupled with significant residual disease in the lymph nodes underlines a more aggressive disease. These data explained the fact that pCR, obtained in both breast and axilla according to Chevallier's classification, was not an independent prognostic factor. Thus, subgroup of patients at different risk of relapse could be selected according to the pathological response in breast and the number of involved nodes. Some authors reported that responders might be good candidates to receive axillary irradiation instead of axillary dissection for local control in the axilla (Lenert *et al*, 1999). In contrast, poor responders to neoadjuvant chemotherapy had a high incidence of histologically positive nodes and are currently best served by axillary dissection. However, axillary dissection remains important not only for nonresponders but also for responders to make decision regarding patient therapy and to improve local control of disease.

In summary, neoadjuvant chemotherapy is able to eradicate any histologic evidence of invasive carcinoma in both the primary breast tumour and axillary lymph nodes in approximately 15% of operable patients. The data indicate that further efforts should focus on elucidating the molecular mechanisms associated with this response. Indeed, only the complete histologic elimination of invasive disease confers a survival advantage; a definitive cure is not warranted and consequently, in absence of pCR, a second non-cross resistant adjuvant regimen or high dose chemotherapy could result in a better patient outcome.
